# FoTeRs: a novel family of telomere-associated retrotransposons in *Fusarium oxysporum*

**DOI:** 10.1186/s13100-025-00385-6

**Published:** 2025-11-24

**Authors:** Sahar Salimi, Mostafa Rahnama

**Affiliations:** 1 School of Environmental Studies, Tennessee Technological University, Cookeville, TN 38505 USA; 2 Department of Biology, Tennessee Technological University, Cookeville, TN 38505 USA

**Keywords:** *Fusarium oxysporum *, Transposable elements (TEs), Retrotransposons, Telomeres, Mutation dynamics, Telomere-linked helicase (TLH)

## Abstract

Transposable elements (TEs) are dynamic components of eukaryotic genomes, playing a crucial role in genome evolution and plasticity, particularly in unstable regions such as chromosome ends. In the globally significant fungal pathogen *Fusarium oxysporum*, we identified and characterized a novel family of non-LTR retrotransposons named FoTeRs (*F. oxysporum *Telomeric Retrotransposons). These elements are consistently and uniquely localized at chromosome ends, representing a rare example of site-specific TE integration. Phylogenetic analysis confirmed that FoTeRs form a distinct clade with other telomere-targeting retrotransposons, suggesting a shared evolutionary history and a convergent mechanism for telomeric integration. We found that individual FoTeR elements exhibit a duality in their evolutionary status. Putatively functional elements are under strong purifying selection, indicating that their protein-coding regions are highly conserved. This contrasts with the presence of other, non-functional copies that exhibit signs of mutational decay, a process accelerated by Repeat-Induced Point (RIP) mutations —a fungal-specific defense mechanism. The high density of upstream variable number tandem repeats (VNTRs) also contributes to their genomic plasticity. Furthermore, FoTeRs frequently co-localize with host Telomere-Linked Helicases (TLHs), suggesting a potential functional link in telomere maintenance. This study provides crucial insights into the role of TEs in shaping the genome architecture and adaptive potential of this important fungal pathogen.

**Supplementary Information:**

The online version contains supplementary material available at 10.1186/s13100-025-00385-6.

## Background

The *Fusarium oxysporum* species complex (FOSC) encompasses a phylogenetically diverse group of fungi with profound impacts on global agriculture and human health [1–3]. While some strains, such as the nonpathogenic *F. oxysporum* Fo47, serve as biocontrol agents against soilborne diseases, others are notorious pathogens capable of infecting a broad spectrum of hosts, including monocotyledonous and dicotyledonous plants, as well as immunocompromised humans and other mammals [2–6]. Despite this wide host range, individual pathogenic isolates exhibit remarkable host specificity, often targeting a single plant species or cultivar. These isolates are classified into distinct formae speciales (abbreviated f. sp., plural ff. spp.), such as *F. oxysporum* f. sp. *fragariae* (strawberry wilt) or f. sp. *lycopersici* (tomato wilt), reflecting their narrow pathogenic niches [5, 7]. While the agricultural devastation caused by these fungi is well-documented, the genetic and genomic mechanisms underlying their host specificity and adaptive evolution remain poorly understood.

Transposable elements (TEs) are increasingly recognized as key drivers of genome evolution and phenotypic diversification in fungi. These mobile genetic elements can move within and between genomic loci, produce mutations, alter gene expression, and reshape chromosome architecture [8, 9]. In *Fusarium* species, TE content varies significantly, correlating with differences in genome structure and pathogenicity. For instance, *F. graminearum* possesses a compact genome with less than 1% TE content, whereas certain *F. oxysporum* strains exhibit TE contents exceeding 17%, primarily within accessory chromosomes associated with host-specific pathogenicity [10, 11]. A particularly dynamic genomic environment for TEs is the subtelomeric region—the chromosome-adjacent domain located between core gene-rich sequences and terminal telomeric repeats. Subtelomeres are gene-poor yet structurally complex, characterized by high recombination rates, tandem repeats, and repetitive DNA blocks [12, 13]. These regions often harbor lineage-specific gene families, virulence-related loci, and secondary metabolite clusters [14, 15]. Their inherent instability and epigenetic regulation make them hotspots of genomic innovation [16, 17]. Due to their unstable nature, high recombination rates, and reduced selective constraints, subtelomeric regions are particularly prone to TE accumulation [18]. In pathogenic fungi, these TEs are frequently positioned near effector genes or within their regulatory regions, where they can modulate expression patterns crucial for virulence and host colonization [19, 20]. For example, telomere-proximal retrotransposons such as the MoTeRs in *Magnaporthe oryzae* have been shown to insert directly into telomeric repeat tracts and induce chromosome end instability [13, 21, 22]. TE insertions in subtelomeres can disrupt nearby coding sequences or induce chromosomal rearrangements, but they can also act as sources of genetic innovation, create novel regulatory elements, foster effector gene diversification, and enable rapid adaptation to host defenses and environmental stressors [23–26].

In this study, we identified a novel group of non-LTR retrotransposons in nine *F. oxysporum* strains, which we designate as FoTeRs (*F. oxysporum* Telomeric Retrotransposons). These elements share structural and sequence similarities with previously characterized MoTeRs and are consistently localized in subtelomeric regions. By comprehensively analyzing their genomic positions, structural organization, distribution across strains, and evolutionary dynamics, we demonstrate that FoTeRs are conserved features of *F. oxysporum* chromosome ends. Our findings provide novel insights into the specific molecular characteristics and evolutionary pressures that shape these elements within *F. oxysporum*, suggesting a potential role for non-LTR retrotransposons in organizing and maintaining telomeric and subtelomeric genome structure.

## Methods

### Genome assemblies

Genome assemblies for five strains of *F. oxysporum* f. sp. *fragariae* (MAFF727510, BRIP62122, GL1080, GL1315, and GL1381), along with *F. oxysporum* f. sp. *cubense* II5, *F. oxysporum* f. sp. *lycopersici* race 3 (Race 3), *F. oxysporum* strain Fo47, and *F. oxysporum* f. sp. *conglutinans* Fo5176, were retrieved from the NCBI database (Table S1A). Although many *F. oxysporum* genomes are publicly available, we selected these nine strains to provide the first comprehensive characterization of this novel element family, focusing on detailed molecular, structural, and evolutionary descriptions of FoTeRs. Genome completeness was assessed using Benchmarking Universal Single-Copy Orthologs (BUSCO) v5.4.7 [27] with the *ascomycota_odb10* dataset consisting of 1,706 conserved genes and Quality Assessment Tool for Genome Assemblies (QUAST) v5.3.0 [28] (Table S1B).

### Refining chromosome end assemblies with sequencing data

Because many chromosome ends in the assemblies were not fully extended to the terminal telomeric repeats, these regions were completed using long-read sequencing data (PacBio). For each strain, PacBio reads (Table S1A) were aligned to the corresponding genome assemblies using minimap2 [29]. The resulting alignments were visually inspected using the Integrative Genomics Viewer (IGV [30]). This step confirmed the current structure of the chromosome ends or, in cases of incomplete assemblies, extended them. The analysis specifically targeted reads spanning from a unique, non-repetitive region nearest the chromosome end to the terminal telomeric repeats. The distance from this unique region to the terminus varied with the length of the repeated non-unique region adjacent to the chromosome ends in the original assemblies. In cases where we found reads that spanned the entire region, these reads—which contained 10 to 30 consecutive TTAGGG telomeric motifs at one end—were included, and the corresponding chromosome ends were then considered complete.

### Identification of FoTeR elements

To identify *Fusarium* telomere-associated retrotransposons (FoTeRs), we used the protein sequence of the *M. oryzae* MoTeR1 reverse transcriptase (RT) open reading frame (ORF) as a query in tBLASTn [31] searches against the genome assemblies of each *F. oxysporum* strain. To ensure the identification of the full element, we extracted a 5 kb flanking region on both sides of the initial tblastn hits. These hits yielded candidate FoTeR elements for each strain, which we aligned with Clustal Omega (version 1.2.3) [32]. We manually inspected the alignments to specify the boundaries of the elements and confirm their telomere association. The resulting multiple sequence alignment is used to generate a consensus sequence for each strain, serving as a reference to define the FoTeRs and identify all genome copies.

We then performed a detailed characterization of the identified elements in each strain. An element was classified as full-length if it met two criteria: first, it was flanked by canonical telomeric repeats at both its 5’ and 3’ ends; and second, it contained a single ORF (detected by the ORF detection function in Geneious Prime (version 25.2.1)) potentially expressing an RT located between these tracts. The longest sequence in each strain that met these two criteria was considered the reference full-length element, especially in cases where multiple copies of the sequence were available, providing stronger evidence for structural completeness, although not a strict requirement when only a single full-length copy existed. A truncated FoTeR, therefore, was defined by its incomplete structure, lacking the full sequence. The structural integrity and accurate delineation of the elements, including the presence of terminal telomeric repeats, were manually verified by inspecting long-read sequencing data (PacBio) aligned to the genome assemblies, which helped resolve any ambiguities.

### Analysis of conserved motifs

For each of the nine *Fusarium* strains examined, putatively functional full-length FoTeR elements were identified (Table 1), and their putative RT ORFs were extracted and translated to proteins. For each strain, a consensus was then generated by aligning these protein sequences using Clustal Omega (version 1.2.3). These consensus protein sequences were then used to identify conserved motifs and domain features using InterProScan [33] with default parameters.

**Table 1:** Summary of FoTeR content and presence in *F*. *oxysporum *strains. This table shows the length of full-length FoTeR elements and their putative ORFs. The total number of FoTeR copies, the total chromosome number for each strain, and the proportion of chromosome ends that contain FoTeR elements for each genome assembly are also provided. For more details, see table S5

	FoTeR length (bp)	FoTeR ORF length (bp)	5’ UTR (bp)	3’ UTR (bp)	No. of FoTeR		Total Chromosome No.	Chromosome Ends Containing FoTeRs (%)
					Full	Truncated		
Fo47	4599	3510	680	409	2	4	12	25.0
Fo5176	4833	3510	914	409	1	20	18	47.2
II5	7285	3555	3366	364	2	18	11	54.5
Race3	4291	3453	492	346	1	3	10	20.0
BRIP62122	6279	3501	2382	396	11	20	11	95.5
MAFF727510	5611	3483	1792	336	3	7	12	41.7
GL1080	4897	3507	1029	361	4	51	14	78.6
GL1315	4456	3504	593	359	5	9	11	63.6
GL1381	5978	3483	2103	392	8	16	14	57.1

### Tandem repeat identification

Variable number tandem repeats (VNTRs) within FoTeR elements were identified using two complementary tools: PHOBOS (version 3.3.12) [34] and Tandem Repeat Finder [35]. PHOBOS was run in imperfect search mode with a repeat unit size range of 6–100 bp, a minimum satellite length of ≥ 6 bp, and up to two successive ambiguous bases (N’s) allowed within a repeat. Imperfections were tolerated using a mismatch score of − 5, a gap score of − 5, and a recursion depth of 5 [34]. Tandem Repeat Finder was executed with parameters 2 7 10 80 10 50 100, where values correspond to match weight, mismatch penalty, indel penalty, match probability, indel probability, minimum alignment score, and maximum period size, respectively. These parameters were chosen to balance sensitivity with specificity, with the higher indel penalty reducing spurious alignments [35]. To ensure reliability, repeats were retained if they were detected by both tools and satisfied the thresholds for minimum repeat length and mismatch tolerance. Motifs with fewer than two repeat units or exceeding the allowed mismatch threshold were discarded as spurious. This analysis was specifically designed to characterize VNTRs internal to FoTeR elements.

The VNTRs were classified based on their motif, length, and genomic location. For each strain, we calculated the total copy number for both shared and strain-specific VNTRs. This calculation includes both full and partial repeat units to accurately reflect the total amount of repetitive sequence. The total length in base pairs (bp) was determined by summing the length of all identified repeats for a given strain.

### Phylogenetics analysis

The consensus RT proteins of FoTeRs generated in the previous section for each *Fusarium* strain were used alongside 95 additional RT-like proteins (Table S2), downloaded from NCBI or translated from corresponding nucleotide sequences when annotated proteins were unavailable. The 95 additional RT-like proteins were selected to provide a broad evolutionary context and to rigorously distinguish FoTeRs from other major retrotransposon families and telomerase reverse transcriptases. Specifically, we included retrotransposons such as Cnl1 (from *Cryptococcus neoformans*), SLACS (from *Trypanosoma*), CRE1/2 (from *Drosophila*), and CZAR (from *Tetrahymena thermophila*), which are known to be site-specific or telomere-associated, as well as canonical LTR and non-LTR retrotransposon families to serve as outgroups. Protein sequences were aligned using Clustal Omega [32], and phylogenetic reconstruction was performed with IQ-TREE2 using the following parameters: sequence type set to amino acids (-st AA), ModelFinder Plus enabled (-m MFP) for automated model selection, 10,000 ultrafast bootstrap replicates (-bb 10000), and an approximate likelihood ratio test with 10,000 replicates (-alrt 10000) [36]. The resulting maximum likelihood tree was visualized using iTOL v6 [37]. A second phylogenetic tree was constructed using only the restriction enzyme-like endonuclease (REL) domains extracted from a representative subset of 20 proteins, including FoTeRs, MoTeR1, and other site-specific retrotransposons. The same alignment and tree-building workflow was applied.

### Identification and classification of FoTeR ORF mutations

For each strain, after aligning the nucleic acid sequences of all detected ORF sequences using Clustal Omega (version 1.2.3) [32], a consensus sequence was generated for the region that represents a full-length ORF sequence. This consensus served as a reference for our mutation study, enabling us to accurately identify and classify mutational dynamics by comparing each individual FoTeR ORF against its strain-specific consensus. A custom Python script was employed to identify mutations through a pairwise comparison of each FoTeR ORF against its corresponding consensus. Detected mutations were meticulously classified into four categories: transitions (G >A, C >T), transversions (A >T, G >C), insertions, and deletions. Strains with only a single FoTeR ORF (Fo5176 and Race3) were excluded from this comparative mutation analysis due to the inability to generate a meaningful within-strain consensus or detect the within-strain variation.

### Analysis of repeat-induced point mutations (RIP)

For each strain, FoTeR ORF sequences were aligned using ClustalW and analyzed with RIPCAL [38] to quantify CpN→TpN substitutions characteristic of RIP. Additionally, custom Python scripts were used to calculate a RIP Product Index, defined as (CA→TA + TG→TA)/(TA→CA + TA→TG), and a RIP Substrate Index, defined as (CpA + TpG)/(ApC + GpT). The transition/transversion ratio (Ti/Tv) and the fraction of C→T transitions at CpA sites were also extracted. FoTeRs were classified as RIP-affected when the RIP Product Index was ≥ 0.75 and the Substrate Index ≤ 1.0, thresholds that distinguish RIP signatures from background mutational noise. Alignments and rolling-window plots generated by RIPCAL were further inspected to confirm the presence of RIP tracts.

### Estimation of substitution rates and selection pressure

To quantify sequence divergence and infer selection pressure on FoTeR RT domains, we focused on FoTeR RT ORFs that were determined to be truly intact. For the purposes of this analysis, an intact ORF (hereafter referred to as ‘intact FoTeR RT ORFs’) was defined as a sequence free of internal stop codons or frameshifts, capable of producing a full, continuous protein product. These sequences were translated into protein using EMBOSS transeq [39]. Multiple sequence alignments of the resulting protein sequences were conducted with MAFFT v7 using the L-INS-i algorithm [40], and this protein alignment was then used to codon-align the corresponding nucleotide sequences via pal2nal.pl to preserve reading frame integrity [41].

Pairwise codon-level nonsynonymous (dN) and synonymous (dS) substitution rates were calculated using the yn00 program from the PAML v4.9 package under the Yang and Nielsen method [42]. Pairwise comparisons were classified as within-strain if both FoTeRs originated from the same genome and between-strain otherwise. Custom Python scripts were used to parse outputs, assign strain-level metadata, and calculate group-wise means.

## Results

### Identifying a novel telomere-associated retrotransposon family

Analysis of the subtelomeric regions across the nine examined *F. oxysporum* species revealed that all strains harbored elements at one or more chromosomal termini. Full-length FoTeR elements range from 4,291 bp to 7,285 bp (Table 1), which include an ORF encoding a putative protein containing a conserved reverse transcriptase (RT) domain. Further investigation, including protein domain comparisons and phylogenetic analysis, confirmed that these elements are additional members of non-LTR retrotransposons, designated as FoTeRs (*F*. *oxysporum* Telomeric Retrotransposons). Notably, no FoTeR elements were detected in other, non-telomeric loci. These elements also exhibited a consistent orientation, with the 5′ end positioned toward the chromosome terminus, similar to the arrangement observed in MoTeR1 elements of *M. oryzae* [21]. Thus, we define the FoTeR family based on a combination of shared structural features, including a consistent domain architecture (RT and REL), their monophyletic clustering in phylogenetic analysis, and their conserved telomere-associated insertion preference.

### FoTeRs possess a conserved non-LTR retrotransposon domain architecture

**Fig. 1:**
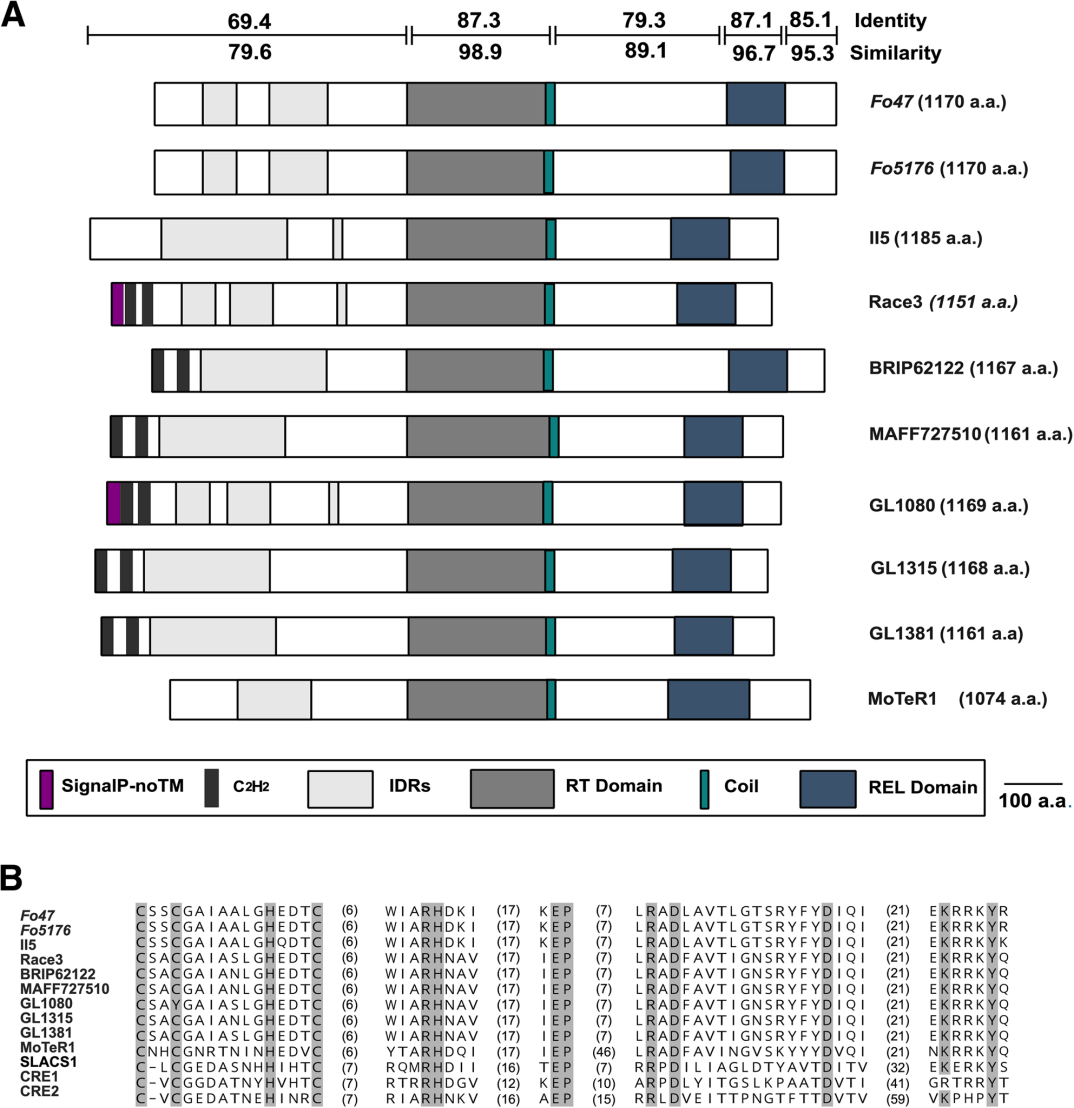
Conserved domain architecture and sequence conservation of FoTeR proteins. **A** Predicted FoTeR proteins from nine *F. oxysporum* strains and MoTeR1 from *Magnaporthe oryzae* were aligned based on their reverse transcriptase (RT) domains. Numbers above and below the bars indicate the average percentage identity and similarity, respectively, calculated for each of the five defined regions (N-terminal, RT domain, inter-domain, REL domain, and C-terminal segments) across all aligned proteins. MoTeR1 is included in the figure as an outgroup for visual context, and its sequence was not considered in the calculation of average identity and similarity values for the FoTeR family. **B** Multiple alignment of restriction endonuclease-like (REL) domains of FoTeR elements, MoTeR1, SLACS1, CRE1, and CRE2. The sequences are segmented into five conserved functional motifs, separated by variable non-conserved spacer regions. The domains shown, arranged from the N-terminus (left) to the C-terminus (right), are: (1) N-terminal CCHC-like Motif, (2) Core REL (RHD/N) Motif, (3) E-motif, (4) Core REL (R/KPD) Motif, and (5) Terminal Motif. Values in parentheses indicate the number of amino acid residues in the non-conserved spacer regions. Amino acid residues highlighted in gray represent the strictly conserved catalytic residues (C, H, D, E) and the adjacent basic/structural residues critical for endonuclease function. The full, unedited alignment is provided as Supplementary Figure S1

Protein domain analysis revealed that the predicted FoTeR proteins consistently contain key reverse transcriptase-associated domains: RT_POL (PROFILE: PS50878, InterPro: IPR000477), RT_dom (InterPro: IPR000477), and RVT_1 (Pfam: PF00078, InterPro: IPR000477), reflecting their functional and evolutionary ties to non-LTR retrotransposons (Fig. 1A). A coiled-coil region (Pfam: PF25600, InterPro: IPR058030) was also detected in all FoTeR proteins, consistently attached to the C-terminus of the RT domain (Fig. 1A). Additionally, all predicted proteins displayed DNA/RNA polymerase superfamily domains, specifically DNA/RNA_pol_sf (InterPro: IPR043502, SUPERFAMILY: SSF56672). C_2_H_2_-type zinc finger motifs (Pfam: PF25420, PF12874, PF13912, and PF00096, InterPro: IPR013087) were also detected in several FoTeR proteins, typically located near the N-terminal region (Fig. 1A). An analysis by the SignalP component of InterProScan predicted a eukaryotic signal peptide in the N-terminal segments of FoTeR proteins from a subset of strains (Race3 and GL1080), an annotation labeled as “SignalP-noTM” (Fig. 1A). This prediction indicates the presence of a signal peptide but the absence of a transmembrane helix. Moreover, intrinsically disordered regions (IDRs) were predicted in the N-terminal segments of multiple FoTeR proteins, indicating the presence of low-complexity or unstructured regions (Fig. 1A).

Further domain characterization revealed a conserved restriction enzyme-like endonuclease (REL-ENDO, hereafter referred to as REL) domain located at the C-terminal region of FoTeR proteins (Fig. 1A). This domain was identified by searching for the REL domain from MoTeR1 of *M. oryzae* (C[X₂]C[X₆]H[X₃]C[X₉]RHD.X₂₀.E.X₄₉.RAD.X₁₂.E) [21], as well as with SLACS (a non-LTR retrotransposon from *Trypanosoma*) and CRE1/2 (a non-LTR retrotransposon from *Drosophila*) (C[X₁-₃]C[X₇-₈]H[X₃-₄]C[X₉-₁₀]RHD/N.X₁₉-₃₃.E.X₉-₂₁.R/KPD.X₁₂-₁₄.D/E) [43]. Our alignment shows that the FoTeR REL domain has a consensus organization of C[X₂]C[X₆]H[X₃]C[X₉]RHD.X₂₀.E.X₁₉.RAD.X₁₂.E, which shares a strong similarity with the MoTeR and SLACS/CRE REL domains (Fig. 1B, Figure S1). Crucially, the alignment confirms that all elements maintain the core REL domain architecture defined by the strictly conserved catalytic residues (C, H, D, E), consistent with a canonical PD-(D/E)XK active site [44–46]. The five motifs delineated in the alignment visually represent this functional core, marked by residues highlighted in gray (Fig. 1B). The N-terminal CCHC-like Motif highlights Cysteine (C) and Histidine (H) residues, which are crucial for forming a zinc-finger-like structure that provides domain stability and likely assists in nucleic acid substrate binding. Following a spacer, the Core REL (RHD/N) Motif features the basic residue Arginine (R), which aids in stabilizing the active site and DNA binding, along with Aspartic acid (D), the first core catalytic residue. We observed intraspecies diversity here, classifying FoTeRs into RHNA-type (Race3, MAFF727510, GL1381, GL1315, GL1080, and BRIP62122, characterized by the ARHNAVN motif within the core REL domain) and RHDK-type (II5, Fo5176, and Fo47 characterized by the ARHDKIV motif within the core REL domain) subgroups; however, MoTeR1 is distinguished by incorporating this residue as part of its specific AD-D variant of the PD-(D/E) motif. The subsequent E-motif highlights Glutamic acid (E), the third strictly conserved catalytic residue vital for metal ion coordination, often adjacent to Proline (P), which is hypothesized to fix the conformation. The Core REL (R/KPD) Motif contains the final Aspartic acid (D) residues, completing the catalytic triad necessary for nuclease function. Finally, the Terminal Motif highlights Lysine (K), which likely stabilizes the DNA-protein complex (the ‘K’ in PD-(D/E)XK), and Tyrosine (Y), suggesting a C-terminal constraint specific to the MoTeR lineage. While this core catalytic architecture is shared, MoTeR1 is clearly differentiated from the FoTeR elements in the third spacer region; the spacer between the E-motif and R/KPD motif in MoTeR1 is much longer (X49) compared to the 7 residues (X7) observed consistently in the FoTeR sequences. These variations reflect distinct evolutionary trajectories even as they maintain the critical architecture for endonuclease activity. These variations in spacer lengths and specific motif subtypes reflect distinct evolutionary trajectories while retaining the core functional architecture necessary for site-specific retrotransposition [47].

To complement the domain architecture analysis, pairwise comparisons among FoTeR variants were conducted across five defined regions—spanning the N-terminal, RT domain, inter-domain region, REL domain, and C-terminal segments. These regions were defined based on the boundaries of the conserved RT and REL domains, with the remaining sequence partitioned as inter-domain and N- and C-terminal segments. These comparisons revealed distinct conservation patterns, with average identity and similarity values calculated for each region based on multiple sequence alignments (Fig. 1A). The reverse transcriptase-associated RT domain exhibited the highest conservation, averaging 87.3% identity and 98.9% similarity, highlighting its critical role in retrotransposon activity. The REL domain showed moderate conservation, with averages of 77.6% identity and 90.0% similarity, retaining strict conservation of catalytic residues despite lower overall sequence identity. Conversely, N-terminal regions, including C_2_H_2_-type zinc finger motifs and IDRs, displayed the lowest conservation, averaging 69.4% identity and 79.6% similarity, likely reflecting structural or functional flexibility in these regions.

### Extensive VNTRs variation highlights genomic heterogeneity of FoTeRs

**Fig. 2:**
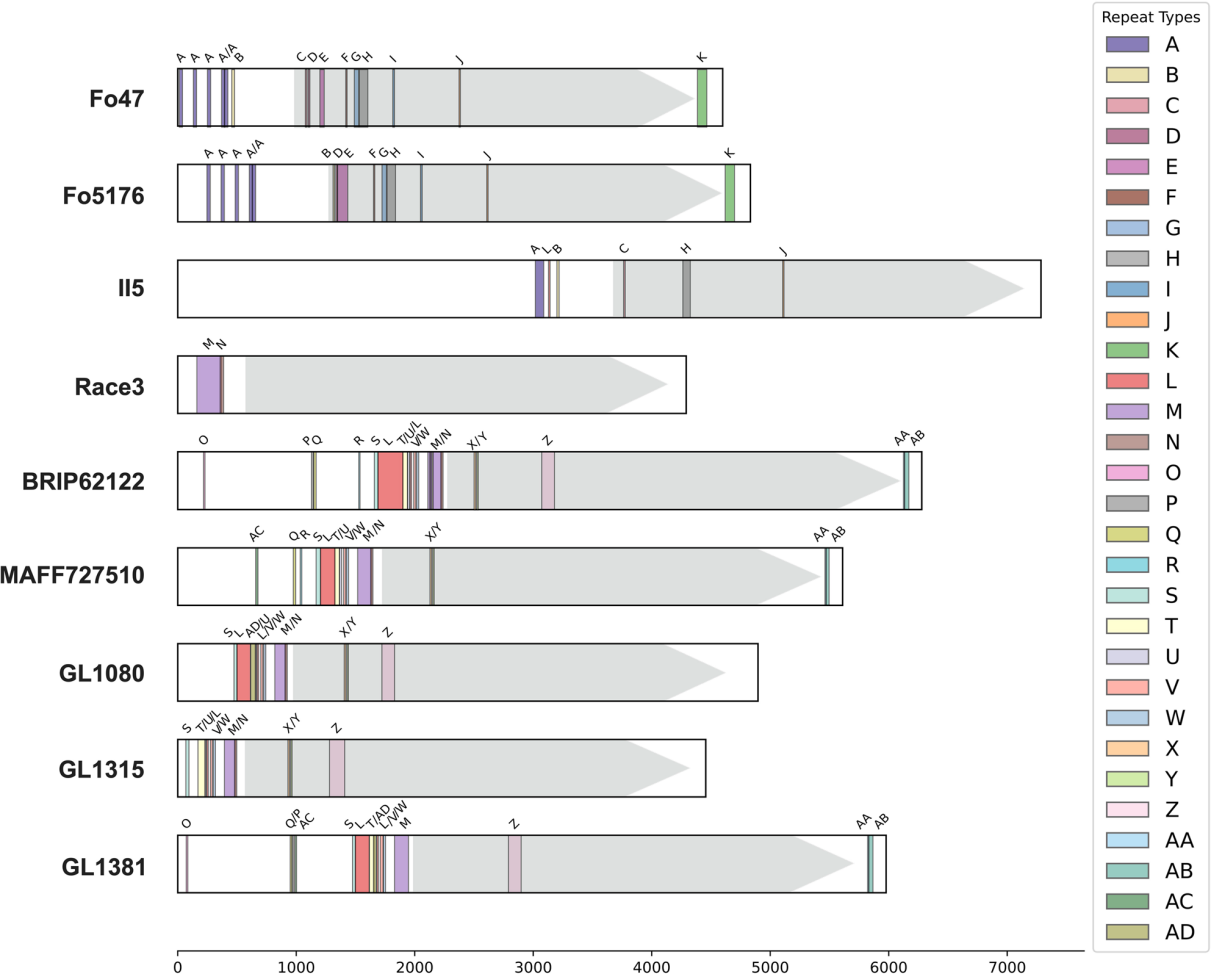
Distribution of shared Variable number tandem repeats (VNTRs) across FoTeR elements. This figure illustrates the positions and distribution patterns of shared VNTR motifs among FoTeR elements identified in *F. orysporum* strains. Each horizontal bar represents a FoTeR element, displaying its RT ORF as a gray arrow-headed box and VNTRs as horizontal blocks whose colors correspond to specific VNTR motifs, as indicated in the legend on the right. VNTR identifiers are labeled above each block. Only VNTRs detected in two or more strains are shown (shared VNTRs). For a comprehensive list of VNTRs and their features, see Table S3

FoTeR elements exhibit notable structural complexity at the nucleotide level, characterized by the presence of multiple variable number tandem repeat (VNTR) motifs (Fig. 2; Table 2). We focused on these repeats because they are a known source of rapid genetic change in pathogens [48]. These VNTRs were predominantly concentrated at the 5′ region of the elements, upstream of the RT ORF, although some were also distributed internally or near the 3′ end.

**Table 2:** Summary of shared and Strain-Specific variable number tandem repeats (VNTRs). This table provides a summary of the VNTRs identified in FoTeR elements from nine *F. oxysporum *strains. VNTRs are categorized as either shared (present in at least two strains) or strain-specific (exclusive to one strain). “Total copy number” column lists the combined number of repeat units per strain, including partial repeats, with the total length in base pairs (bp) in parentheses. “Identifiers” column lists the designated labels for each repeat, with a subscript indicating the number of times each identifier occurs. For the complete dataset, see table S3

	**Shared VNTRs**			**Strain-Specific VNTRs**	
	**Number **	**Identifiers**	**Total copy number (length)**	**Number **	**Total copy number (length)**
**Fo47**	15	A_5_, B, C, D, E, F, G, H, I, J, K	41.6 (461 bp)	2	6.1 (152 bp)
**Fo5176**	14	A_5_, B, D, E, F, G, H, I, J, K	38.9 (415 bp)	5	13.1 (230 bp)
**II5**	6	A, L, B, C, H, J	17.1 (194 bp)	12	28.6 (472 bp)
**Race3**	2	M, N	12.3 (238 bp)	5	15.2 (187 bp)
**BRIP62122**	18	O, P, Q, R, S, L_2_, T, U, V, W, M, X, Y, N, Z, AA, AB	67.7 (743 bp)	7	22.6 (603 bp)
**MAFF727510**	15	AC, Q, R, S, L, T, U, V, W, M, N, X, Y, AA, AB	52.3 (521 bp)	6	24.6 (553 bp)
**GL1080**	12	S, L_2_, AD, U, V, W, M, N, X, Y, Z	45.4 (496 bp)	4	10.0 (382 bp)
**GL1315**	11	S, T, U, L, V, W, M, N, X, Y, Z	34.8 (421 bp)	3	15.0 (219 bp)
**GL1381**	15	O, Q, P, AC, S, L_2_, T, AD, V, W, M, Z, AA, AB	54.6 (602 bp)	15	39.1 (793 bp)
**MoTeR1**	1	F	2 (12 bp)	16	69.2 (1167 bp)

VNTR motifs identified within FoTeR elements were categorized into two groups: shared VNTRs, which occurred in two or more strains, and strain-specific (SS) VNTRs, which were unique to individual genomes. A total of 33 shared VNTR motifs were identified across the dataset, along with 64 SS motifs, yielding 97 distinct VNTR sequences overall (Table S3). The total copy number, which included both full and partial repeats, also varied substantially between strains. Among all strains, GL1381 exhibited the highest VNTR coverage, with shared and SS motifs spanning 1395 bp of its FoTeR length, while Race3 showed the lowest VNTR coverage at just 425 bp.

The number and composition of VNTRs varied substantially between strains. Shared VNTRs ranged from 2 in Race3 to 18 in BRIP62122. The total number of shared VNTR copies was highest in GL1381 (54.6) and BRIP62122 (67.7), covering 602 bp and 743 bp of the FoTeR length, respectively. In contrast, Race3 and II5 exhibited the lowest levels of shared VNTR content, with only 12.3 and 17.1 copies, corresponding to 238 bp and 194 bp of their respective FoTeR elements.

Strain-specific (SS) VNTRs also contributed notably to the total repeat content of FoTeRs. GL1381 carried 15 SS motifs totaling 39.1 copies and covering 793 bp of the element. Similarly, II5 and MAFF727510 harbored 12 and 6 SS motifs (28.6 and 24.6 copies), accounting for 472 bp and 553 bp, respectively. In contrast, Fo5176 and Race3 contained relatively few SS repeats (5 each), with limited span (230 bp and 187 bp, respectively).

The data also revealed notable internal variability of VNTR copies within a single strain. As detailed in Table S3, some VNTR identifiers (e.g., ‘A’ in Fo47) appear at multiple genomic locations with different copy numbers and lengths. This observation highlights the genomic heterogeneity of VNTRs and, importantly, suggests an active process of repeat expansion and contraction. These results suggest that such dynamic VNTR activity may play a direct role in facilitating genetic plasticity in *F. oxysporum*, potentially influencing its adaptation and evolution.

Mapping VNTR locations against ORF coordinates revealed a consistent positional trend: most VNTRs were clustered upstream of the RT ORF, with several occurring in intergenic or untranslated regions (Fig. 2). For example, in Fo47, the RT ORF spanned nucleotides 681–4190, while nearly all shared VNTRs were located before position 1800, reinforcing their enrichment near the 5′ end. Similar upstream biases were observed in GL1315 (ORF: 594–4097), GL1080 (1030–4536), and MAFF727510 (1793–5275), although in strains like II5 and BRIP62122, some VNTRs extended within or slightly downstream of the ORF.

In comparison, the VNTR structure of the MoTeR1 element is distinct from that observed in FoTeRs, both in composition and location. Our analysis revealed only one shared VNTR motif between MoTeR1 and the FoTeR elements (Table 2). The remaining 16 VNTR motifs identified in MoTeR1 were all strain-specific and were not found in any of the *F. oxysporum* strains examined. Furthermore, while FoTeR VNTRs are concentrated predominantly in the 5’ region, the MoTeR1 VNTRs are distributed more broadly. The MoTeR1 element contains a large cluster of strain-specific VNTRs at its extreme 5’ end (spanning the first ~ 2,000 bp), but also includes repeats in its 3’ region (SS13-SS16), with the largest tandem repeat array (SS14) located near the 3’ end (Table S3). This contrasts with the FoTeR elements, where VNTRs are overwhelmingly biased toward the 5’ region, with very few located at the 3’ end.

### FoTeRs belong to a conserved clade of telomere-targeting retrotransposons

**Fig. 3:**
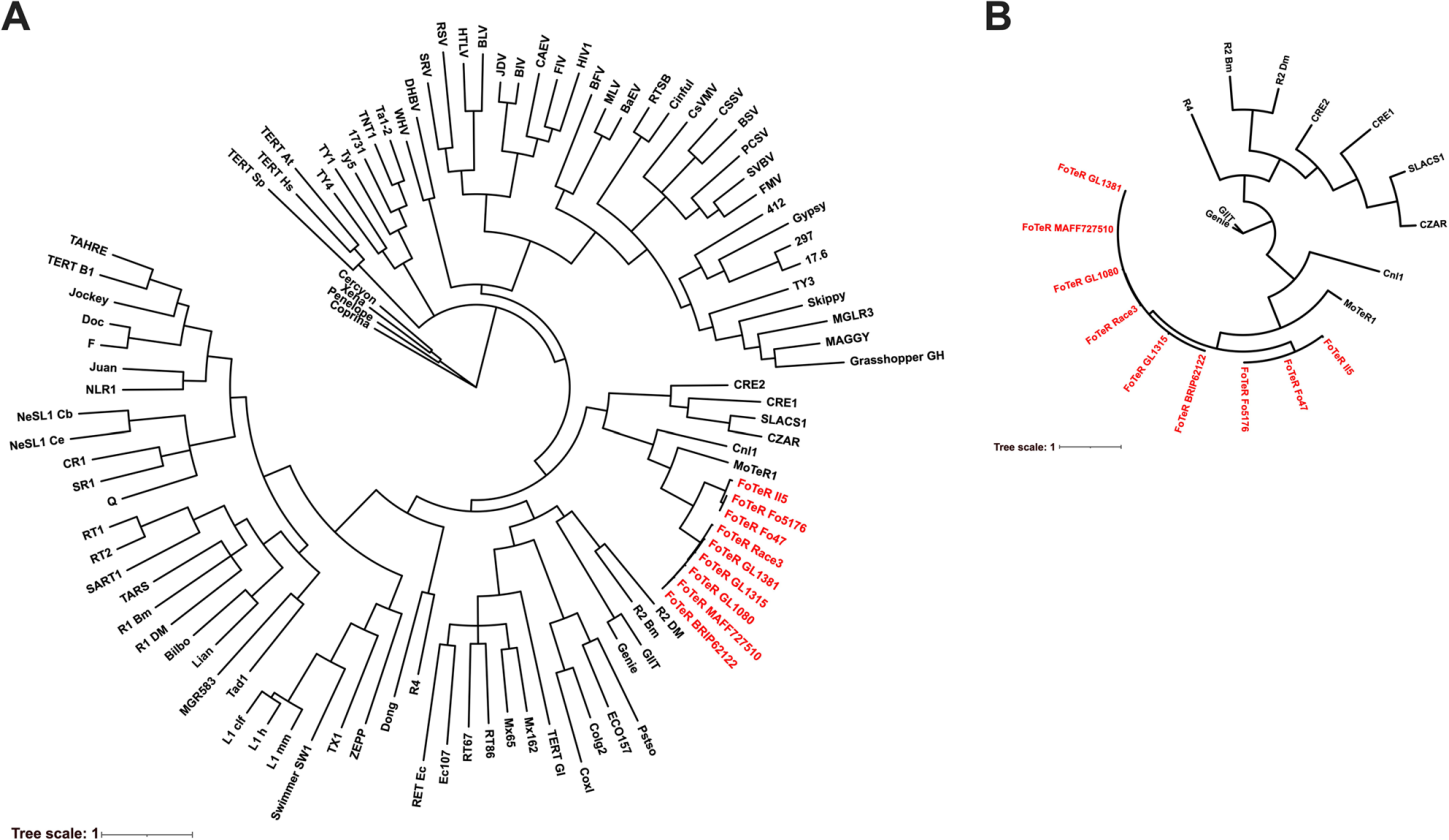
Phylogenetic placement of FoTeRs based on reverse transcriptase (RT) protein and REL domain. **A** Unrooted maximum likelihood tree of FoTeR RT proteins (consensus full-length ORF translations from nine *F. oxysporum *strains), alongside 95 additional RT-like proteins from various retrotransposon families (Table S2). **B** Unrooted phylogenetic tree of restriction endonuclease-like (REL) domains from a representative subset of 20 retrotransposons, including FoTeRs, MoTeR1, and other site-specific elements. In both trees, branch support was assessed using 10,000 ultrafast bootstrap replicates; branches with <50% support were collapsed. FoTeR elements are highlighted in red

Phylogenetic analyses of FoTeR RT proteins positioned these elements within a well-supported, monophyletic fungal clade that includes MoTeR1, Cnl1, SLACS, CRE1/2, and CZAR homologs (Fig. 3A; see also Supplementary Figure S2A). The FoTeR clade comprises two distinct subgroups: one consisting of Race3, MAFF727510, GL1381, GL1315, GL1080, and BRIP62122, and the other consisting of II5, Fo5176, and Fo47. The deliberate inclusion of diverse outgroups, such as canonical LTR retrotransposons and TERTs, allowed us to formally confirm that FoTeRs are a novel family of non-LTR retrotransposons. FoTeRs branched distinctly from protist-derived non-LTR retrotransposons such as *Giardia* Genie (GilT) and R2 elements, which occupied basal positions outside the fungal telomeric clade. Canonical retrotransposons, including Gypsy, Jockey, and L1 families, as well as telomerase reverse transcriptases from humans, *Saccharomyces pombe*, and *Arabidopsis thaliana*, formed independent, distantly related lineages, reinforcing the unique evolutionary trajectory of the FoTeR-containing group [9, 21]. The clustering of FoTeRs within a well-supported fungal clade, separate from these other major families, confirms their identity as a novel non-LTR family. This clade, which also includes MoTeR1, Cnl1, SLACS, CRE1/2, and CZAR, encompasses retrotransposons previously characterized as site-specific or telomere associated. Across the nine *F. oxysporum* strains, FoTeR consensus proteins exhibited high sequence similarity and clustered tightly together, indicating limited divergence and strong conservation across species boundaries.

To further resolve the relationships among site-specific retrotransposons, we constructed a separate maximum likelihood tree using only the REL domains from a subset of 20 representative elements. This analysis recovered a similar topology, with FoTeRs clustering tightly with MoTeR1, Cnl1, SLACS, CRE1/2, and CZAR (Fig. 3B; Supplementary Figure S2B). As in the RT-based tree, telomeric elements were grouped with robust support. The consistency across both RT and REL domain phylogenies supports the evolutionary cohesion of this group and reinforces their shared telomeric localization and potential functional similarity in site-specific integration.

### Asymmetric junctions suggest TPRT-mediated telomere integration

To understand the mechanism by which FoTeRs integrate into the genome, we examined their insertion junctions, specifically looking for evidence of target-primed reverse transcription (TPRT), a common integration mechanism for non-LTR retrotransposons [47]. Such mechanisms often result in Target Site Duplications (TSDs), where the host DNA sequence is duplicated at the insertion site [47]. Analysis of 5′ and 3′ junctions from nine *Fusarium* strains revealed that FoTeRs are consistently associated with telomeric or telomere-like sequences. At the 5′ ends, elements were flanked by short telomeric motifs, which varied slightly between strains and included both canonical sequences (TAACCC) and degenerate variants (e.g., CCTAAC, AACCCT, CCCTAA, CCAATC). These motifs were observed once per junction in most strains, except in MAFF727510 and GL1315, which each contained two copies (Table 3 and Table S4). Notably, a conserved 5′ flanking sequence adjacent to the telomeric motif was consistently identified across all FoTeR copies within each strain (Table 3). In contrast, the 3′ ends of all FoTeRs were followed by multiple copies of the canonical *Fusarium* telomeric repeat TAACCC, typically ranging from 3.0 to 7.0 copies.

**Table 3:** Summary of FoTeR integration junctions and flanking sequences. The telomeric repeats and flanking sequences that are conserved across all FoTeR copies within each *F*. *oxysporum *strain are presented. Asterisks (*) in columns detailing telomeric repeats of truncated FoTeRs (tFoTeRs) indicate that all tFoTeRs identified in that strain were truncated at the respective end, so there is no telomeric association with that end

	Intact FoTeRs			tFoTeRs	
Strain	5’ Flanking Sequence (Copy No.)	5’ Telomeric Repeat (Copy No.)	3’ Telomeric Repeat (Copy No.)	5’ Telomeric Repeat (Copy No.)	3’ Telomeric Repeat (Copy No.)
Fo47	AATAGT (1)	ACCTA (1)	TAACCC (3.5–4.0)	*	TAACCC (4.5)
Fo5176	ACTAGC (1)	TAACCC (1)	TAACCC (3.1)	TAACCC (1)	TAACCC (3.4–4.3)
II5	GTCTGT (1)	TAACC (1)	TAACCC (3.3–3.6)	TAACC (1)	TAACCC (3.0–3.8)
Race3	TTTGGA (1)	CCAATC (1)	TAACCC (4.3)	CCAATC (1)	*
BRIP62122	CAATCA (2)	AACCCT (1)	TAACCC (3.5–6.0)	*	TAACCC (4.0)
MAFF727510	CACTTG (1)	TAACCC (2)	TAACCC (3.0–4.0)	TAACCC (2)	TAACCC (3.0–8.0)
GL1080	TAATAA (1)	CCTAAC (1)	TAACCC (3.0–6.0)	CCTAAC (1)	TAACCC (4.0–4.5)
GL1315	CAAAAT (1)	TAACCC (2)	TAACCC (3.3–6.3)	TAACCC (2)	TAACCC (3.0–4.3)
GL1381	ACCCAA (3–10)	CCCTAA (1)	TAACCC (3.2–7)	*	TAACCC (3.0–4.2)

Due to the highly repetitive nature of the telomeres, we were unable to definitively identify TSDs. The presence of telomeric motifs at both ends is consistent with telomere-associated insertion, but these flanking repeats are not classified as TSDs. Instead, these repeats are an inherent part of the telomeric sequence where the retrotransposon has inserted. The asymmetry between the 5’ and 3’ ends suggests FoTeR integration likely involves a TPRT mechanism. Consistent with this, in truncated FoTeRs, the side opposite to the truncation always retained terminal telomeric repeats (Table 3).

### Co-localization of FoTeRs with telomere-linked helicases (TLHs)

**Fig. 4:**
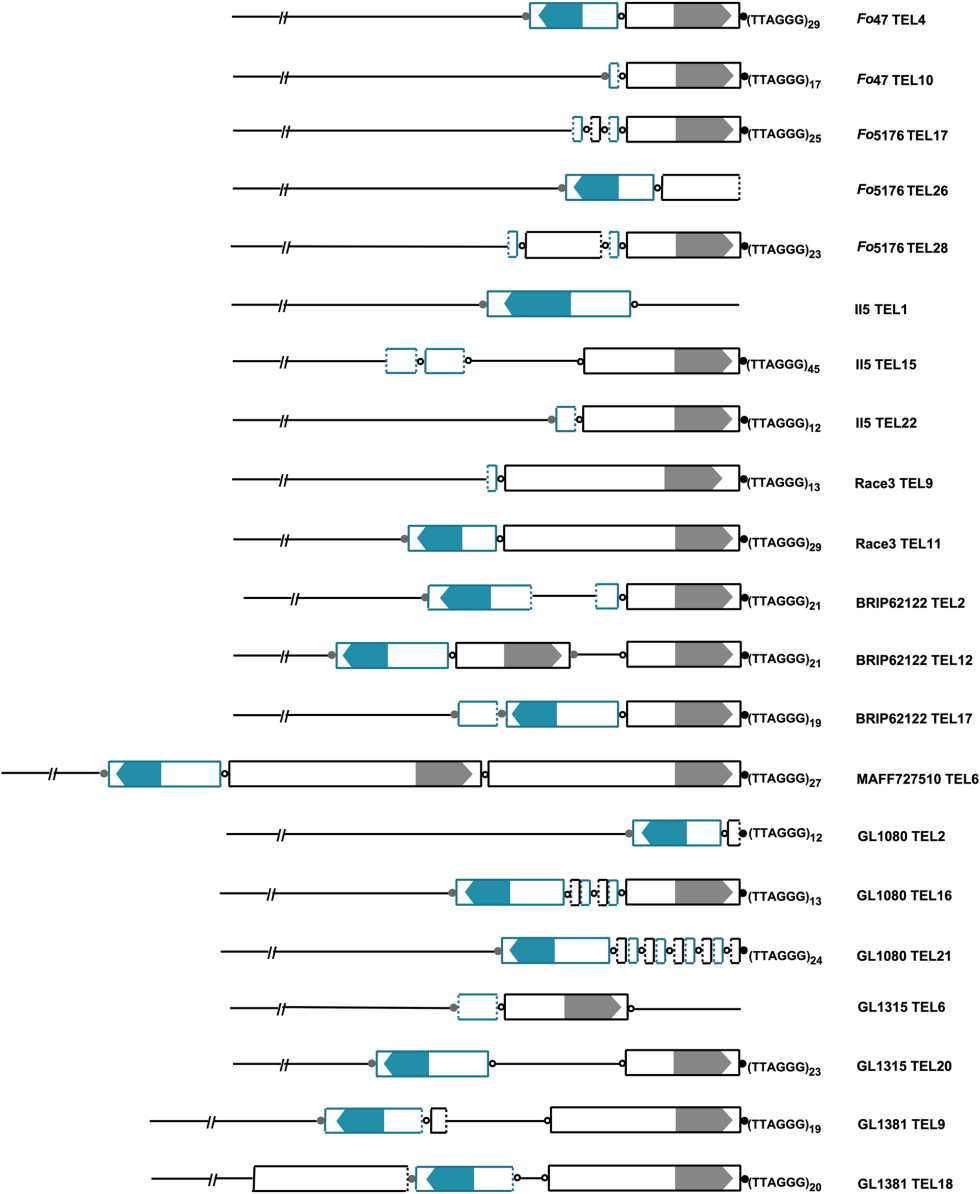
Examples of FoTeRs organization and distribution in the chromosome ends of *F*. *oxysporum *strains. Each horizontal line represents a chromosome end, with the corresponding strain name indicated on the right. Within these regions, gray rectangular boxes illustrate *tlh*-containing regions (TLHcrs), with *tlh* ORFs marked by gray arrows. Dark blue rectangular boxes represent FoTeR elements, with their Reverse Transcriptase (RT) ORFs marked by dark blue arrows. Boxes (both gray and dark blue) having dotted angles denote truncated forms of TLHcrs or FoTeRs, respectively. Solid black circles symbolize terminal telomere repeats. Open circles indicate interstitial telomere repeats with only one repeat, whereas gray circles show interstitial telomeres with more than one repeat. For detailed data, see Table 1 and Table S5

FoTeR element lengths exhibited considerable variation across strains, ranging from a minimum of 4,291 bp in strain Race3 to a maximum of 7,285 bp in II5. Despite this overall length variability, the FoTeRs’ ORF was relatively conserved across strains, ranging from 3,453 bp to 3,555 bp. An examination of nine *F. oxysporum* strains revealed that FoTeRs are widespread but vary notably in copy number and chromosomal distribution (Fig. 4; Table 1; Supplementary Table S5). The total number of FoTeRs per genome ranged from 4 (comprising 1 full-length and 3 truncated copies) in Race3 to 55 (comprising 4 full-length and 51 truncated copies) in GL1080 (Table 1; Supplementary Table S5). In all examined strains, truncated FoTeR copies consistently outnumbered full-length elements.

The proportion of chromosome ends containing FoTeRs also varied substantially, ranging from 20.0% (4 of 10 total ends) in Race3 to 95.5% (21 of 22 total ends) in BRIP62122 (Table 1).

Telomere-linked helicases (TLHs) [49] were frequently found in association with FoTeRs across all nine *Fusarium* strains. In most cases, FoTeRs co-occurred with TLHs at chromosome ends, suggesting a potential structural or functional linkage (Table 4; Fig. 4). Strains with higher overall TLH counts, such as Fo47 (24 TLHs), BRIP62122 (22 TLHs), GL1080 (37 TLHs), and GL1381 (24 TLHs), generally exhibited a greater number of FoTeRs and a higher proportion of chromosome ends containing both elements (Tables 1 and 4).

**Table 4:** Genomic characteristics of *Tlh* genes and TLHcr (*tlh*-containing regions) in* F*. *oxysporum *strains, summarizing their length characteristics, copy numbers, and co-localization patterns with FoTeR elements at chromosome ends

	TLHcr length (bp)	tlh ORF length (bp)	No. of TLHcrs		No. of Chr ends with		
			Full	Truncated	Only FoTeR	Only TLH	FoTeR & TLH
Fo47	4599	3510	22	2	0	18	6
Fo5176	4833	3510	13	5	0	0	15
II5	7285	3555	6	8	3	5	9
Race3	4291	3453	4	15	0	14	4
BRIP62122	6279	3501	21	1	1	1	21
MAFF727510	5611	3483	16	18	1	12	8
GL1080	4897	3507	17	20	0	0	22
GL1315	4456	3504	9	11	0	5	11
GL1381	5978	3483	14	10	0	2	19

Across strains, chromosome ends fell into three distinct categories based on TLH and FoTeR presence: ends with both TLH and FoTeR, ends with only TLH, and ends with only FoTeR. For instance, in GL1080 and BRIP62122, nearly all FoTeR-containing chromosome ends also harbored TLHs, with 22 and 21 ends, respectively, showing co-occurrence (Table 4). In contrast, strains like MAFF727510 and Race3 had a notable number of ends with TLHs alone (12 and 14 ends, respectively), indicating that TLHs are also capable of localizing independently of FoTeRs. Ends with only FoTeRs were relatively rare, observed in only a few strains, such as II5 (3 ends) and MAFF727510 (1 end) (Table 4). These patterns highlight a common but not obligatory linkage between TLHs and FoTeRs at *Fusarium* telomeres, suggesting overlapping but distinct telomeric roles or recruitment mechanisms for these elements.

### Sequence divergence and mutation dynamics across FoTeR ORFs

To evaluate sequence conservation and mutational variation within the FoTeR ORFs across *F. oxysporum* strains, a detailed analysis of mutation dynamics was performed. The analysis included all 53 identified FoTeR elements that have a sequence representing a full-length ORF (regardless of whether internal stop codons or mutations prevent the production of a functional full-length protein). Two strains, Race3 and Fo5176, each contained only a single full-length FoTeR ORF and were therefore excluded from the comparative mutation analysis, as within-strain comparisons to a consensus sequence were not feasible. Among the remaining seven strains, the number of analyzed FoTeR ORFs ranged from 2 (Fo47) to 17 (GL1381) (Table 5). The overall pairwise identity values among FoTeR ORFs within these strains spanned from 87.96% (II5) to 100% (GL1381). The total number of mutations detected per strain and the average mutation load per ORF varied considerably. For instance, Fo47, with only two copies, exhibited an exceptionally low average of 4 mutations per ORF, whereas II5, with only 3 copies, displayed a substantially higher average of 143 mutations per ORF (Table 5). In contrast, GL1381, with 17 copies, showed a high total number of mutations (n = 339), but a lower average mutation load per ORF (41.4), highlighting a complex relationship between copy number and mutation load. Transition mutations were the dominant type of base substitution across most strains, consistent with common replication errors or deamination processes [50]. The highest total counts of transitions were observed in BRIP62122 (n = 346) and GL1381 (n = 339) (Table 5). These transitions, particularly G >A and C >T changes, are characteristic of replication-associated or deamination-driven processes. Transversion mutations were also detected, though generally at lower frequencies, with the highest count observed in GL1381 (n = 63) (Table 5 and Table S6). Notably, no transitions or transversions were detected in Fo47 (n = 0 for both), while II5 exhibited a few transitions (n = 4) but no transversions, highlighting distinct nucleotide substitution patterns in these two strains (Table 5). Insertions and deletions (indels) contributed substantially to the overall mutational burden in several genomes. BRIP62122 showed the highest number of insertions (n = 412), followed by GL1381 (n = 145). Conversely, II5 displayed extensive deletion activity (n = 425), with GL1080 also showing a considerable number of deletions (n= 211) (Table 5).

**Table 5:** Context of mutational events in FoTeR ORFs of *F. oxysporum* strains. The mutation analysis includes all FoTeR elements that have a sequence representative of a full-size ORF. Detailed comparisons are provided in table S6

	Number of FoTeRs	Pairwise identity range (%)	Average mutations per ORF	Mutations			
				Transition	Transversion	Insertion	Deletion
Fo47	2	99.9–99.9	4.0	0	0	8	0
II5	3	87.9–99.9	143.0	4	0	0	425
BRIP62122	13	96.0–99.2	82.8	346	45	412	273
MAFF727510	5	97.6–99.4	32.0	78	28	54	0
GL1080	7	95.4–99.4	67.0	198	40	20	211
GL1315	4	95.4–98.2	52.5	73	25	93	19
GL1381	17	96.0–100.0	41.4	339	63	145	156

To assess the potential influence of RIP, a fungal-specific defense mechanism against repeats, we analyzed the FoTeR ORFs for characteristic G: C to A: T transition mutations. Our analysis, which was performed on FoTeR sequences from seven genomes, revealed that five isolates (Fo47, BRIP62122, GL1080, GL1315, and GL1381) exhibited a clear signal of RIP (Table 6). Conversely, two isolates (II5 and MAFF727510) lacked a clear RIP signature. The results of this analysis provide evidence that RIP is an evolutionary force shaping the mutational landscape of FoTeR elements in a strain-specific manner.

**Table 6:** Evidence of RIP signatures across *F. oxysporum* isolates. Ti/Tv = transition/transversion ratio. RIP product and substrate indices represent enrichment of RIP-associated dinucleotide mutations and depletion of susceptible sites, respectively. CpA < sub > frac_trans</sub > indicates the fraction of C→T transitions occurring at CpA sites. The combined evidence (“RIP Call”) denotes whether each isolate exhibits hallmarks of RIP (RIP product and substrate indices ≥ 0.75 and ≤ 1.0, respectively, in combination with elevated CpA-associated C→T transitions)

Isolate	Ti/Tv Ratio	RIP Product Index	RIP Substrate Index	CpA_frac_trans	RIP Call
Fo47	–	1.00	1.00	0.50	RIP
II5	–	–	–	0.60	NotRIP
BRIP62122	6.33	0.75	0.75	0.50	RIP
MAFF727510	3.38	0.64	0.64	0.37	NotRIP
GL1080	4.64	1.51	1.51	0.51	RIP
GL1315	3.90	1.50	1.50	0.37	RIP
GL1381	3.85	1.13	1.13	0.49	RIP

### Selective constraints on functional FoTeR RT

While many FoTeR ORFs (24 of 53) exhibit mutational decay, a substantial portion remains intact and potentially able to produce a functional protein. To assess selective pressures acting on the putatively functional FoTeR RT proteins, a dN/dS analysis was performed on 29 intact FoTeR RT sequences. A total of 401 valid pairwise comparisons were analyzed after filtering for complete dN/dS data. These comparisons were categorized into 69 within-strain comparisons and 332 between-strain comparisons. Across all analyzed comparisons, the average dN/dS ratio was consistently less than 1, indicating that the functional FoTeR RT domains are predominantly under purifying (negative) selection. This suggests that non-synonymous mutations that alter the amino acid sequence are generally deleterious and are removed by natural selection, thereby preserving the functional integrity of the RT domain. For within-strain comparisons (n= 69), the mean dN was 0.008 (standard deviation [SD]: 0.030), the mean dS was 0.042 (SD: 0.135), and the mean dN/dS ratio was 0.174 (SD: 0.072). The dN/dS ratios for within-strain comparisons ranged from 0.000 to 0.500. In contrast, between-strain comparisons (n= 332) exhibited higher levels of sequence divergence for dS, with a mean dN of 0.064 (SD: 0.104), a mean dS of 0.311 (SD: 0.487), and a mean dN/dS ratio of 0.179 (SD: 0.051) (Table S7A). The dN/dS ratios for between-strain comparisons ranged from 0.084 to 0.429. The raw pairwise dN, dS, and dN/dS values for all comparisons are provided in Supplementary Table S7B.

**Fig. 5:**
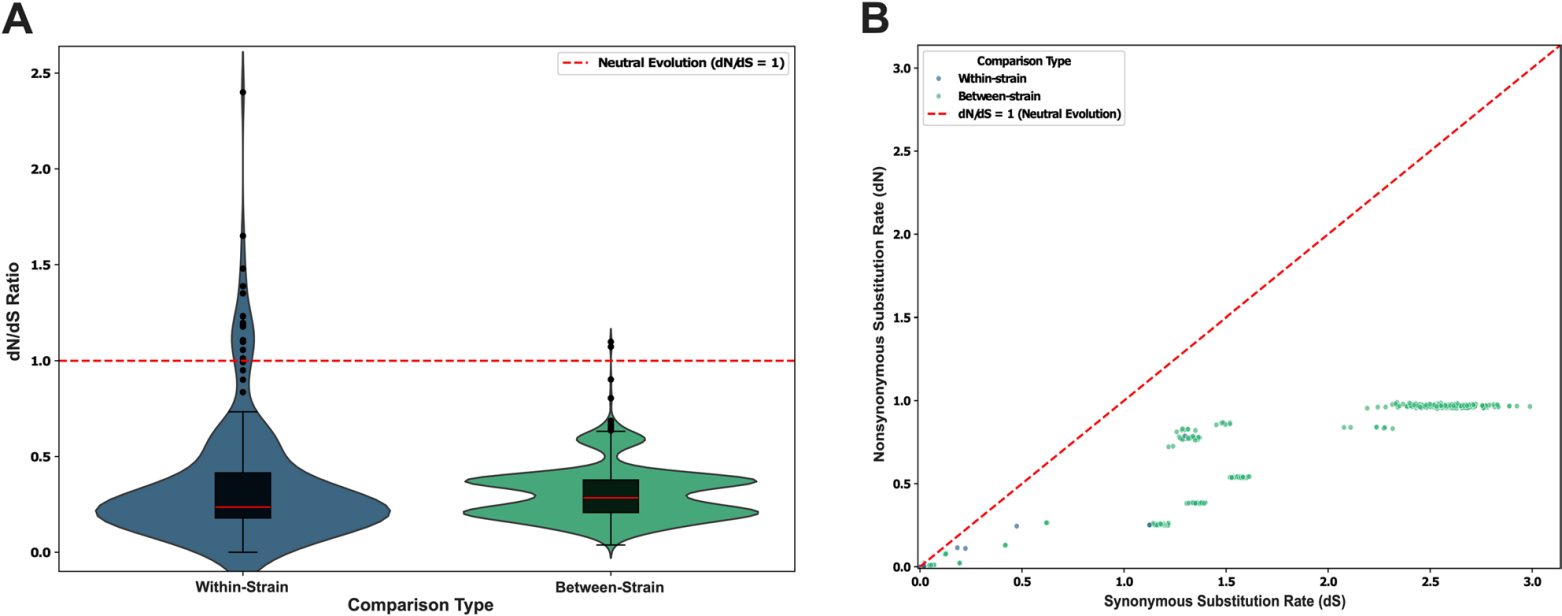
Analysis of selective pressures on FoTeR RT protein.** A** Combined violin and box plot of dN/dS ratios. Distribution of dN/dS values for within-strain (n = 96) and between-strain (n = 332) pairwise comparisons. The violin plot represents the kernel density estimate of the data, while the overlaid box plot indicates the median (red horizontal line), interquartile range (box), and 1.5× interquartile range (whiskers), with individual points representing outliers. A dashed red horizontal line at dN/dS = 1 denotes the expectation under neutral evolution. **B** Scatter plot of synonymous (dS) versus nonsynonymous (dN) substitution rates. Each point represents a single pairwise comparison between FoTeR RT sequences. Points are colored according to their comparison type (within-strain or between-strain). The dashed red diagonal line indicates the dN/dS = 1 ratio, where dN equals dS

The distributions of these dN/dS ratios are visually represented in Fig. 5 and Figure S3, illustrating the density and frequency of dN/dS values for both within- and between-strain comparisons. Notably, 12 pairwise comparisons (all within-strain) yielded a dN/dS ratio of exactly 0, indicating complete conservation at the amino acid level for these specific pairs (dN = 0, dS > 0). The relationship between synonymous and nonsynonymous substitution rates across all pairwise comparisons is further depicted in Fig. 5B, where each point represents a comparison colored by its type.

While our analysis revealed consistent structural features, telomeric association, and strain-specific variation of FoTeR elements across nine *F. oxysporum* genomes, these findings may not fully represent the entire diversity of FoTeRs within the *F. oxysporum* species complex. Given the remarkable genomic variability of this group, additional analyses across a broader range of isolates will be required to determine how generalizable these patterns are. This study therefore provides a foundational framework for understanding FoTeR biology and evolution, upon which future large-scale and automated comparative studies can build to explore their broader diversity and functional significance.

## Discussion

Transposable elements are pervasive and dynamic components of eukaryotic genomes, contributing significantly to genome evolution, plasticity, and adaptation [51, 52]. Their persistence within host genomes often represents a precarious balance between their inherent capacity for replication and the host’s intricate mechanisms of suppression and mutational decay [53, 54]. Our comprehensive analysis of FoTeRs in *F. oxysporum* sheds light on the interplay of these evolutionary forces, examining both the selective pressures maintaining putatively functional elements and the broader mutational dynamics affecting the entire FoTeR population.

Our characterization of FoTeRs reveals a novel family of non-LTR retrotransposons that integrate specifically at chromosome termini, similar to the well-studied MoTeR1 elements described in *M. oryzae* [21]. Such site-specificity is a rare but intriguing characteristic among retrotransposons, placing FoTeRs within a small, specialized group that preferentially targets repetitive chromosomal loci [21, 55, 56]. A key distinction of these elements is that while many other TEs are found in subtelomeric regions, our analysis indicates that FoTeRs are the only retrotransposons detected within the telomeric sequences themselves. The presence of putatively functional ORFs under strong purifying selection suggests that the FoTeR population is not merely a collection of old, non-active remnants, but also includes elements that are maintained by selection. This heterogeneity is further supported by the presence of two distinct subgroups, which we have designated as RHNA-type and RHDK-type elements, highlighting the intraspecies diversity of these elements. The question of whether a TE insertion creates a repetitive locus or if a TE prefers an existing one is a compelling question that we leave for future research.

Structurally, FoTeRs strikingly mirror MoTeR1, encoding a large open reading frame (approximately 1,150–1,184 amino acids) containing both a conserved RT domain and a distinct REL domain while conspicuously lacking LTRs (Fig. 1A) [21]. Integration is likely mediated by a target-primed reverse transcription (TPRT) mechanism, a common mode for non-LTR retrotransposons, in which the endonuclease activity of the REL domain precisely cleaves telomeric DNA to initiate cDNA synthesis [57]. The presence of a canonical PD-(D/E)XK active site within the FoTeR REL domain (Fig. 1B) further aligns FoTeRs with well-studied site-specific elements such as SLACS and CRE1/2 [44–46]. Our detailed analysis of FoTeR insertion junctions confirmed their consistent association with telomeric sequences and revealed a structural asymmetry between the 5’ and 3’ ends (Table 3; Supplementary Table S4). While definitive target site duplications (TSDs) could not be identified due to the repetitive nature of the telomere, the consistent appearance of telomeric motifs at both ends strongly supports telomere-associated insertion, a pattern consistent with TPRT-mediated integration. This observation, and the absence of a detectable TSD, may indicate that these insertions are ancient and conserved. This suggests FoTeR integration likely involves mechanisms such as microhomology-mediated repair or nonhomologous end joining, similar to what has been proposed for MoTeRs [13, 21, 22].

The evolutionary relationship of FoTeRs to other site-specific retrotransposons is further elucidated by phylogenetic analysis. The inclusion of diverse outgroups, such as LTR retrotransposons and TERTs, was crucial for this analysis, as it formally confirms the identity of FoTeRs as a novel non-LTR family. Both FoTeR RT and REL protein sequences consistently cluster within a well-supported, monophyletic fungal clade that includes MoTeR1, Cnl1, SLACS, CRE1/2, and CZAR homologs (Fig. 3A, B; Supplementary Figure S2A, B). This robust phylogenetic placement is a foundational finding of our study, distinguishing FoTeRs from other major mobile genetic elements and highlighting their unique evolutionary trajectory. Importantly, the RT protein phylogeny also reveals that the FoTeR clade comprises two distinct subgroups. This clustering, however, does not perfectly align with the host strain phylogeny, suggesting a complex evolutionary history for these elements that is not a straightforward reflection of host divergence. The independent emergence of telomere-targeted retrotransposons across diverse eukaryotic lineages [58], including this fungal-specific clade, represents a compelling instance of convergent evolution, highlighting an adaptive advantage of telomeric localization for TE persistence.

Our dN/dS analysis specifically evaluated the selective pressures acting on the putatively functional FoTeR RT domains, representing those elements with intact open reading frames capable of encoding a full protein. This analysis revealed a consistent pattern of strong purifying (negative) selection, with mean dN/dS ratios of 0.344 for within-strain comparisons and 0.303 for between-strain comparisons. These values are substantially below 1, indicating that amino acid changes in the RT domain are generally deleterious and are effectively removed by natural selection. This strong purifying selection is characteristic of functionally important protein-coding regions and strongly suggests that these putatively functional FoTeR RT domains are under continuous evolutionary constraint, likely reflecting their ongoing activity in retrotransposition or other critical roles within the *F. oxysporum* genome. The notable incidence of pairwise comparisons with dN/dS ratios of exactly 0 (Table S7A) further highlights regions or copies under extreme conservation. The similar dN/dS ratios across both within- and between-strain comparisons imply that the fundamental selective pressures maintaining the integrity of functional FoTeR RT domains are broadly consistent across different levels of divergence and distinct *F. oxysporum* lineages. This suggests a conserved functional requirement for the RT enzyme, irrespective of the specific host genetic background or evolutionary time. However, it is important to note that this dN/dS analysis, by its nature, focused exclusively on putatively functional ORFs and thus does not directly capture selection pressures on degenerating or non-coding TE copies [42]. Further phylogenetic approaches to dN/dS could be employed to identify site-specific or branch-specific selection pressures and reconstruct more precise evolutionary histories.

Complementing the dN/dS analysis, our investigation into the mutation dynamics across all 53 FoTeR ORF regions (including those that may not encode a full, continuous protein) revealed a diverse landscape of mutational processes (Table 5, Table S6). The considerable variation in total mutation load and average mutations per ORF across strains (ranging from 4 in Fo47 to 143 in II5) underscores the heterogeneous evolutionary trajectories among FoTeR populations. Transition mutations were broadly dominant consistent with the typical spontaneous mutational biases often observed in fungal genomes [50], which may be driven by replication errors or deamination events. A more detailed analysis of the dominant transition mutations revealed a clear signal of RIP in some strains, supporting the hypothesis that these transitions result from this fungal-specific defense mechanism. The striking absence of base substitutions in Fo47, coupled with an exceptionally low total mutation load, suggests a remarkably conserved FoTeR population in this strain, potentially indicative of very recent insertion events or particularly efficient purifying selection acting even at the nucleotide level. While our consensus-based mutation analysis provides a detailed snapshot of nucleotide and indel variation relative to a representative sequence, it does not infer ancestral states. Crucially, insertions and deletions (indels) emerged as significant contributors to the overall mutational burden in several FoTeR populations with extensive deletion activity observed in strains such as II5 and GL1080. This suggests processes of progressive decay and pseudogenization in these copies, contrasting sharply with the purifying selection observed in the putatively functional FoTeR RTs. This dual observation of strong conservation in functional elements and widespread decay in others is a common theme in TE evolution, where TEs lacking active transposition machinery accumulate mutations more readily [59]. The presence of a RIP signal in some FoTeR elements offers new insights into the multifaceted evolutionary pressures that shape these elements. While purifying selection acts to maintain the integrity of putatively functional FoTeRs, RIP acts as a host defense mechanism against repetitive sequences, contributing to the mutational decay observed in the population. This interplay between purifying selection and host-driven mutation provides a strong explanation for the dual evolutionary patterns we observed [60].

FoTeR elements also display extensive structural complexity at the nucleotide level due to the accumulation of variable number tandem repeat (VNTR) motifs (Fig. 2; Table 2; Table S3). Our analysis of these VNTRs is crucial because they are a primary source of rapid genetic change, which can drive adaptation and virulence in pathogens [48]. The heterogeneity and discontinuity of the VNTR motifs are not a result of random coincidence but rather a signature of non-random evolutionary processes. This is supported by their presence as shared sequences across multiple FoTeRs, indicating a common origin. Furthermore, the motifs are not randomly distributed, but are consistently concentrated in the 5’ region, suggesting a specific structural or functional role. This non-random positional bias, combined with the presence of both conserved and unique (strain-specific) motifs, indicates that the observed heterogeneity is the result of non-random biological processes like replication slippage and recombination, which drive repeat expansion and contraction. This dynamic activity may directly contribute to genetic plasticity in *F. oxysporum* by enabling genomic changes that could facilitate its adaptation and evolution [21, 61]. VNTR-rich regions are well known to promote replication slippage, recombination, and secondary structure formation, all of which could contribute to element instability or variation in expression potential [48, 62]. This pattern is consistent with that observed in MoTeR elements [21], although a detailed comparison reveals that while both elements utilize VNTRs, their evolutionary strategies differ. The presence of shared VNTR motifs among FoTeR elements across different *F. oxysporum* strains suggests a degree of ancestral conservation, indicating that some of these repeats have been maintained over time. In contrast, MoTeR1’s repeat content is overwhelmingly defined by highly divergent, strain-specific motifs. This distinction suggests that while telomere targeting is a convergent strategy, the specific genomic features and evolutionary dynamics of these TEs are unique to each lineage. The differences in VNTR composition and positional bias—with FoTeRs clustering repeats almost exclusively at the 5’ end and MoTeRs having large arrays at both ends—underscore the diverse ways in which retrotransposons can use repetitive sequences to potentially influence their own function and stability, which in turn contributes to the overall genomic plasticity and adaptation of their host [48, 62].

Our findings also highlight the complex genomic distribution of FoTeRs and their notable association with Telomere-Linked Helicases (TLHs). FoTeR element lengths span a wide range, and truncated copies consistently outnumber full-length elements in all examined strains, suggesting ongoing processes of degradation or incomplete retrotransposition [22, 52]. FoTeRs are found at a high proportion of chromosome ends, and TLHs are frequently co-localized (Table 4; Fig. 4). This frequent association of FoTeRs and TLHs at chromosome termini suggests a potential structural or functional linkage, where TLHs may play a role in telomere maintenance that indirectly influences FoTeR dynamics or vice versa. However, observing some chromosome ends with only TLHs, or only FoTeRs indicates that, while they often co-occur, their localization is not strictly interdependent, suggesting overlapping but distinct telomeric roles or recruitment mechanisms. This pattern is a particularly compelling observation, as a similar association is not observed between MoTeRs and TLHs in *M. oryzae* [13, 63]. This suggests a potentially distinct relationship in FoTeR dynamics with TLHs in *F. oxysporum* compared to the association of MoTeRs with TLHs in *M. oryzae*. It is speculated that telomere-associated tandem repeats may be important for the expression or regulation of telomere-linked genes, including TLHs, providing a fascinating parallel to the co-localization observed for FoTeRs [49]. Investigating the specific host factors and environmental conditions that might contribute to the observed strain-specific differences in FoTeR copy number, mutation accumulation, and degradation rates would provide a more complete understanding of their dynamic interplay with the *F. oxysporum* genome.

## Conclusions

Our study provides a comprehensive genomic and evolutionary analysis of FoTeRs, a novel family of telomere-associated non-LTR retrotransposons in *F. oxysporum*. We demonstrate their unique structural and phylogenetic relationship to other site-specific retrotransposons, highlighting their definitive preference for telomeric integration. In addition, our findings reveal strong purifying selection acting on their putatively functional RT domains, indicating vital functional constraints on active elements. Concurrently, we characterize a dynamic landscape of mutational decay, marked by the accumulation of various substitutions and indels, particularly evident in non-functional copies. The complex interplay observed with TLHs and the strain-specific variations in FoTeR populations underscore the multifaceted evolutionary pressures shaping these elements and suggest their potential involvement in telomere dynamics and genome plasticity within the host. This research advances our understanding of a new family of non-LTR retrotransposon elements in fungal genomes, and we believe that future experimental validation of RT activity will be a compelling avenue to directly link sequence data to biological function.

### Data Availability

The data supporting this study are from both public and non-public sources. Complete FoTeR element sequences and custom codes are in a public GitHub repository at https://github.com/RahnamaLab/FoTeR_Project.git. A subset of sequences is available in the NCBI GenBank database, listed by accession number (Table S1). Raw PacBio sequencing reads referenced in Table S1 were provided by other laboratories and are not publicly available; however, they can be obtained upon reasonable request from the original providers.

## Supplementary Information

Supplementary Material 1. Table S1. Genomic resources and genome completeness of *F. oxysporum* strains used in this study. A) Lists the strains analyzed along with their data sources. B) Results of BUSCO genome completeness analysis based on 1,706 conserved fungal genes from the fungi_odb10 dataset

Supplementary Material 2: Table S2. Protein sequences used in the phylogenetic analysis of FoTeRs.

Supplementary Material 3. Table S3. Tandem repeats identified within FoTeR elements across *F. oxysporum* strains. Both shared and strain-specific variable number tandem repeats (VNTRs) are included for each strain, detailing the repeat identifier, the full tandem repeat sequence, its genomic location, and the number of copies. The table also includes internal subheadings to distinguish between shared and strain-specific VNTRs for each strain.

Supplementary Material 4. Table S4. Detailed FoTeR integration site information and flanking sequence architecture. This table illustrates the structural organization of FoTeR and truncated FoTeR (tFoTeR) insertions at chromosomal ends of each strain, detailing strand-specific junction features, including flanking repeat sequences, adjacent telomeric repeats, and copy number estimates for both 5′ and 3′ ends of FoTeR insertions. Repeat sequences are annotated with motif identity and approximate tandem copy number; for example, 'TAACCC/3.5' indicates the motif 'TAACCC' is repeated 3.5 times. The status of each FoTeR element (full-length or truncated) is displayed in the column 'FoTeR/tFoTeR.' Full-length FoTeR elements are designated as 'FoTeR.' Truncated FoTeRs (tFoTeRs) are denoted by their genomic coordinates within parentheses, where the coordinates indicate the start and end positions of the identified truncated element. For example, (1-145) signifies a tFoTeR truncated from its 3' end at location 145 bp, while (4494-6201) indicates a tFoTeR truncated from its 5' end at location 4494 bp. Asterisks (*) in columns detailing telomeric repeats of tFoTeRs indicate that tFoTeR/s in that chromosome end in that strain were truncated at the respective end, so there is no telomeric association with that end.

Supplementary Material 5. Table S5. A comprehensive overview of the characteristics of each chromosome's end, including details on the numbers of identified FoTeRs and TLHs.

Supplementary Material 6. Table S6. Mutational analysis of FoTeR ORFs across *F. oxysporum* strains. A) Extended details of Table 5 show strain-specific mutation profiles across FoTeR ORFs. B–H) Strain-specific alignments of FoTeR ORFs from the following strains: B) II5, C) BRIP62122, D) GL1080, E) GL1315, F) GL1381, G) MAFF727510, and H) Fo47. The FoTeR ORF sequences from each strain were aligned using ClustalOmega to detect mutations compared to its consensus sequence. Only nucleotide positions with at least one mismatch are shown; conserved positions are omitted for clarity. Unmutated positions (matching the consensus sequence) are denoted by periods, and a '-' sign indicates deletions. Naming of FoTeRs is associated with the telomere number. For example, FoTeR 9 means this FoTeR is located in TEL9 (the start of chromosome 5).

Supplementary Material 7. Table S7. Pairwise dN, dS, and dN/dS values for FoTeR-RT ORF sequences within and between *F. oxysporum* strains. A) Summary statistics of pairwise dN/dS comparisons among intact FoTeR ORFs. B) Detailed pairwise comparisons showing the raw dN, dS, and dN/dS values for each pair of sequences.

Supplementary Material 8. Figure S1. Multiple sequence alignment of FoTeR REL domains with MoTeR1, SLACS1, and CRE1/2. This figure shows the complete, unedited alignment of the restriction endonuclease-like (REL) domains used in this study.

Supplementary Material 9. Figure S2. Alternate layout of phylogenetic trees of FoTeRs and related retrotransposons. (A) Maximum likelihood tree of FoTeR RT proteins (consensus full-length ORF translations from nine *F. oxysporum* strains) alongside 95 RT-like proteins from various retrotransposon families (Table S2), displayed in rectangular layout. (B) Maximum likelihood tree of restriction endonuclease-like (REL) domains from 20 representative retrotransposons, including FoTeRs, MoTeR1, and other site-specific elements, displayed in rectangular layout. In both trees, branch support was assessed using 10,000 ultrafast bootstrap replicates; branches with <50% support were collapsed. FoTeR elements are highlighted in red.

Supplementary Material 10. Figure S3. Distribution of dN/dS ratios. Histograms illustrating the frequency distribution of dN/dS ratios for within-strain and between-strain comparisons. Kernel density estimates are overlaid to show the distribution shape. A dashed red vertical line at dN/dS=1 indicates the threshold for neutral evolution.

## Data Availability

The data supporting this study are from both public and non-public sources. Complete FoTeR element sequences and custom codes are in a public GitHub repository at https://github.com/RahnamaLab/FoTeR_Project.git. A subset of sequences is available in the NCBI GenBank database, listed by accession number (Table S1). Raw PacBio sequencing reads referenced in Table S1 were provided by other laboratories and are not publicly available; however, they can be obtained upon reasonable request from the original providers.
