# Supplementary material for: FoTeRs: a novel family of telomere-associated retrotransposons in Fusarium oxysporum
Source: Mob DNA. 2025 Nov 24;17:5. doi: 10.1186/s13100-025-00385-6 (PMC12857005; doi:10.1186/s13100-025-00385-6)
Supplement: Supplementary file 1 — Supplementary Material 1. Table S1. Genomic resources and genome completeness of F. oxysporum strains used in this study. A) Lists the strains analyzed along with their data sources. B) Results of BUSCO genome completeness analysis based on 1,706 conserved fungal genes from the fungi_odb10 dataset [file 13100_2025_385_MOESM1_ESM.pdf]

Table S1. Genomic resources and genome completeness of *F. oxysporum* strains used in this study. A) Lists the strains analyzed along with their data sources.

Strain Name	Strain's Abbreviation	GenBank Assembly Number	PacBio Reads
Fusarium oxysporum Fo47	**Fo47**	GCA_013085055.1	SRR11526760
Fusarium oxysporum f. sp. *conglutinans* Fo5176	**Fo5176**	GCA_030345115.1	SRR22746921
Fusarium oxysporum f. sp. *Cubense* II5	**II5**	GCA_031834405.1	From Li Jun Ma (UMass Amherst)
Fusarium oxysporum f. sp. *Lycopersici* race3	**Race3**	GCA_003977725.1	SRR7892102
Fusarium oxysporum f. sp. *fragariae* BRIP62122a	**BRIP62122**	GCA_016166325.2	From Peter Henry (USDA)
Fusarium oxysporum f. sp. *fragariae* MAFF727510	**MAFF727510**	GCA_016164145.2	From Peter Henry (USDA)
Fusarium oxysporum f. sp. *fragariae* GL1080	**GL1080**	GCA_016170085.2	SRR10386447
Fusarium oxysporum f. sp. *fragariae* GL1315	**GL1315**	GCA_016166095.2	From Peter Henry (USDA)
Fusarium oxysporum f. sp. *fragariae* GL1381	**GL1381**	GCA_016170095.2	SRR10313629

B) Results of BUSCO genome completeness analysis based on 1,706 conserved fungal genes from the fungi_odb10 dataset.

Isolate	Complete BUSCO (%)	Complete and single-copy BUSCOs (%)	Complete and duplicated BUSCOs (%)	Fragmented BUSCOs (%)	Missing BUSCOs (%)
**Fo47**	98.0	97.1	0.9	0.1	1.9
**Fo5176**	98.2	94.3	3.9	0.1	1.7
**II5**	98.2	97.7	0.5	0.2	1.6
**Race3**	97.7	97.3	0.4	0.4	1.9
**BRIP62122**	98.3	97.7	0.6	0.2	1.5
**MAFF727510**	95.0	94.4	0.6	0.2	4.8
**GL1080**	98.1	97.2	0.9	0.2	1.7
**GL1315**	98.0	97.2	0.8	0.2	1.8
**GL1381**	97.9	97.1	0.8	0.3	1.8
