# Supplementary material for: FoTeRs: a novel family of telomere-associated retrotransposons in Fusarium oxysporum
Source: Mob DNA. 2025 Nov 24;17:5. doi: 10.1186/s13100-025-00385-6 (PMC12857005; doi:10.1186/s13100-025-00385-6)
Supplement: Supplementary file 2 — Supplementary Material 2: Table S2. Protein sequences used in the phylogenetic analysis of FoTeRs. [file 13100_2025_385_MOESM2_ESM.pdf]

Table S2. Protein sequences used in the phylogenetic analysis of FoTeRs.

Name	Organism	Genbank Accession
Bilbo	*Drosophila subobscura*	AAB92389.1
CR1	*Gallus gallus*	AAA49027.1
Doc	*Drosophila melanogaster*	CAA35587.1
F	*Drosophila melanogaster*	AAA28508.1
Jockey	*Drosophila funebris*	P21329.1
Juan	*Aedes aegypti*	AAA29354.1
Lian	*Aedes aegypti*	AAB65093.1
MGR583	*Magnaporthe grisea*	AAB71689.1
NeSL1(Cb)	*Caenorhabditis briggsae*	AAZ15238.1
NeSL1(Ce)	*Caenorhabditis elegans*	T25782
NLR1	*Chironomus thummi*	AAB26437.2
Q	*Anopheles gambiae*	AAA53489.1
R1(Bm)	*Bombyx mori*	AAC13649.1
R1(Dm)	*Drosophila melanogaster*	P16425.1
RT1	*Anopheles gambiae*	AAA29363.1
RT2	*Anopheles gambiae*	AAA29365.1
SART1	*Bombyx mori*	BAA19776.1
SR1	*Schistosoma mansoni*	AAC06263.1
Tad1	*Neurospora crassa*	AAA21792.1
TAHRE	*Drosophila melanogaster*	CAD65869.1
TERT_B1	*Drosophila melanogaster*	T13173
TRAS	*Bombyx mori*	BAA07467.1
L1(clf)	*Canis lupus familiaris*	BAA25253.1
L1(h)	*Homo sapiens*	AAC51276.1
L1(mm)	*Mus musculus*	P11369.2
R2(Bm)	*Bombyx mori*	T18197
R2(Dm)	*Drosophila mercatorum*	AAB94032.1
R4	*Ascaris lumbricoides*	S60004
Swimmer_SW1	*Oryzias latipes*	AAD02928.1
TX1	*Xenopus laevis*	P14381.1
ZEPP	*Chlorella vulgaris*	BAA25763.1
Cnl1	*Cryptococcus neoformans* var. neoformans JEC21	XP_567259.1
CRE2	*Crithidia fasciculata*	AAB40036.1
CRE1	*Crithidia fasciculata*	AAA75435.2
CZAR	*Trypanosoma cruzi*	B41950
Genie	*Giardia intestinalis*	AAL76330.1
GIIT	*Giardia intestinalis*	ABB04054.1
MoTER1	*Magnaporthe oryzae*	AFI41156.1
SLACS1	*Trypanosoma brucei*	XP_827644.1

Colg2	mitochondrion *Podospora anserine*	CAA38781.1
CoxI	mitochondrion *Marchantia polymorpha*	AAC09454.1
Ec107	*Escherichia coli*	CAA44468.1
ECO157	*Escherichia* coli O157:H7 str. Sakai]	NP_052642.1
Mx162	*Myxococcus xanthus*	P23072.1
Mx65	*Myxococcus xanthus*	P23071.1
Pstso	chloroplast *Scenedesmus obliquus*	P19593.1
RET(Ec)	*Escherichia coli*	CAA78293.1
RT67	*Escherichia coli*	P21325.2
RT86	*Escherichia coli*	P23070.1
1731	*Drosophila melanogaster*	S00954
Ta1_2	*Arabidopsis thaliana*	CAA37920.1
TNT1	*Nicotiana tabacum*	P10978.1
TY1	*Saccharomyces cerevisiae*	P47100.3
Ty4	*Saccharomyces cerevisiae*	P47024.3
Ty5	*Saccharomyces paradoxus*	AAC02631.1
Grasshopper_GH	*Magnaporthe oryzae*	AAA21442.1
MAGGY	*Magnaporthe oryzae*	AAA33420.1
MGLR3	*Magnaporthe oryzae*	AAK01619.1
RETRO61	*Magnaporthe oryzae*	
Skippy	*Fusarium oxysporum*	S60179
17.6	*Drosophila melanogaster*	P04323.1
297	*Drosophila melanogaster*	P20825.1
412	*Drosophila melanogaster*	P10394.1
Cinful	*Zea mays*	AAD11615.1
Gypsy	*Drosophila melanogaster*	P10401.1
RTSb	*Sorghum bicolor*	AAD27571.1
TY3	*Saccharomyces cerevisiae*	Q7LHG5.2
BSV	*Banana streak OL virus*	NP_569150.1
CSSV	*Cacao swollen shoot virus*	NP_041734.1
CsVMV	*Cassava vein mosaic virus*	NP_056848.1
FMV	*Figwort mosaic virus*	NP_619548.1
PCSV	*Peanut chlorotic streak virus*	NP_042513.1
SVBV	*Strawberry vein banding virus*	NP_043933.1
BFV	*Bovine foamy virus*	NP_044929.1
BaEV	*Baboon endogenous virus* strain M7	P10272.2
BIV	*Bovine immunodeficiency virus* R29	P19560.2
BLV	*Bovine leukemia virus*	P25059.2
CAEV	*Caprine arthritis encephalitis virus* strain Cork	P33459.1
FIV	*Feline immunodeficiency virus (isolate TM2)*	P31822.1
HIV1	*Human immunodeficiency virus* type 1 (MAL ISOLATE)	P04588.3
HTLV	*Human T--cell lymphotrophic virus type 1 (strain ATK)*	P03362.3
JDV	*Jembrana disease virus*	Q82851.1
MLV	AKR (endogenous) *murine leukemia virus*	P03356.3

RSV	*Rous sarcoma virus -- Prague C*	P03354.2
SRV	*Simian retrovirus 1*	P04025.2
DHBV	*Duck hepatitis B virus* strain China	P30028.1
WHV	*Woodchuck hepatitis virus* 59	P12899.1
TERT(At)	*Arabidopsis thaliana*	AAD54276.1
TERT(Gl)	*Giardia lamblia ATCC 50803*	XP_001709571.1
TERT(Hs)	*Homo sapiens* (human)	NP_937983.2
TERT(Sp)	*Schizosaccharomyces pombe*	AAC49803.1
Cercyon	*Schistosoma mansoni*	DAA00890.1
Coprina	*Phanerochaete chrysosporium*	AAX11377.1
Penelope	*Drosophila virilis*	AAA92124.2
Xena	*Takifugu rubripes (Fugu rubripes)*	AAK58879.1
