# Supplementary figures and images for: FoTeRs: a novel family of telomere-associated retrotransposons in Fusarium oxysporum
Source: Mob DNA. 2025 Nov 24;17:5. doi: 10.1186/s13100-025-00385-6 (PMC12857005; doi:10.1186/s13100-025-00385-6)

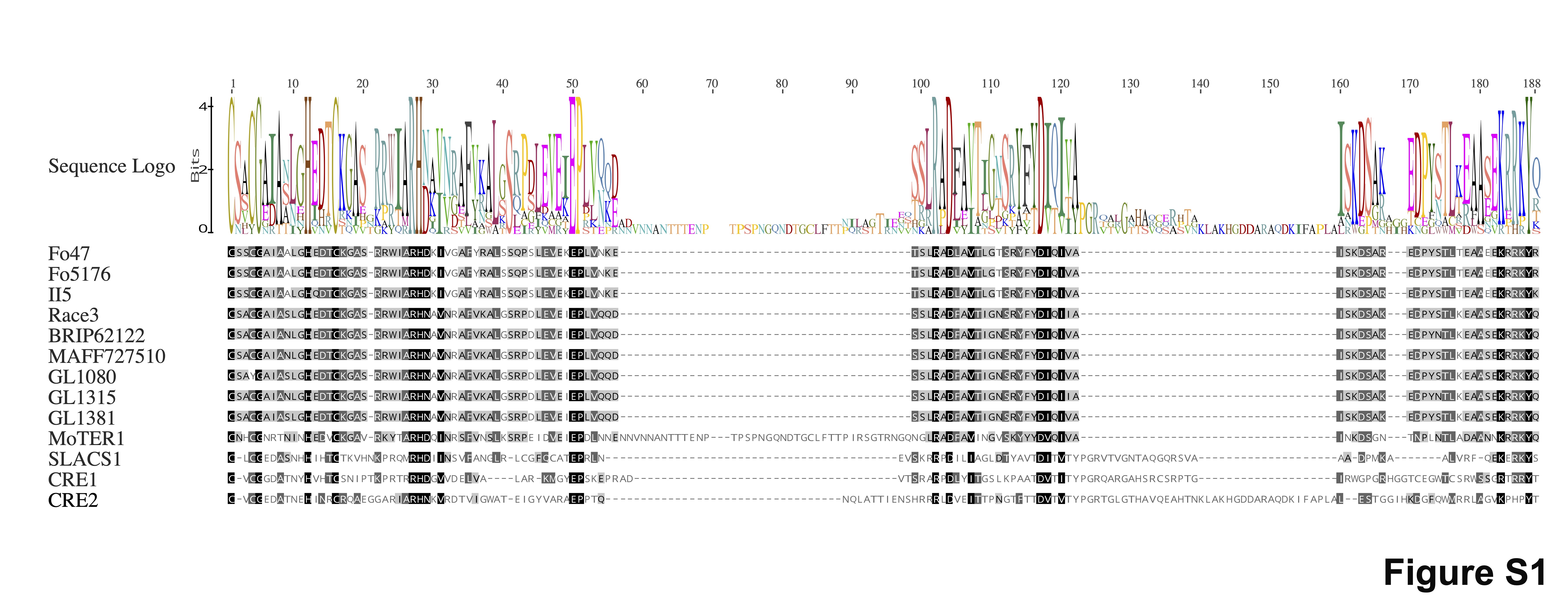

Supplement: Supplementary file 8 — Supplementary Material 8. Figure S1. Multiple sequence alignment of FoTeR REL domains with MoTeR1, SLACS1, and CRE1/2. This figure shows the complete, unedited alignment of the restriction endonuclease-like (REL) domains used in this study. [file 13100_2025_385_MOESM8_ESM.jpg]

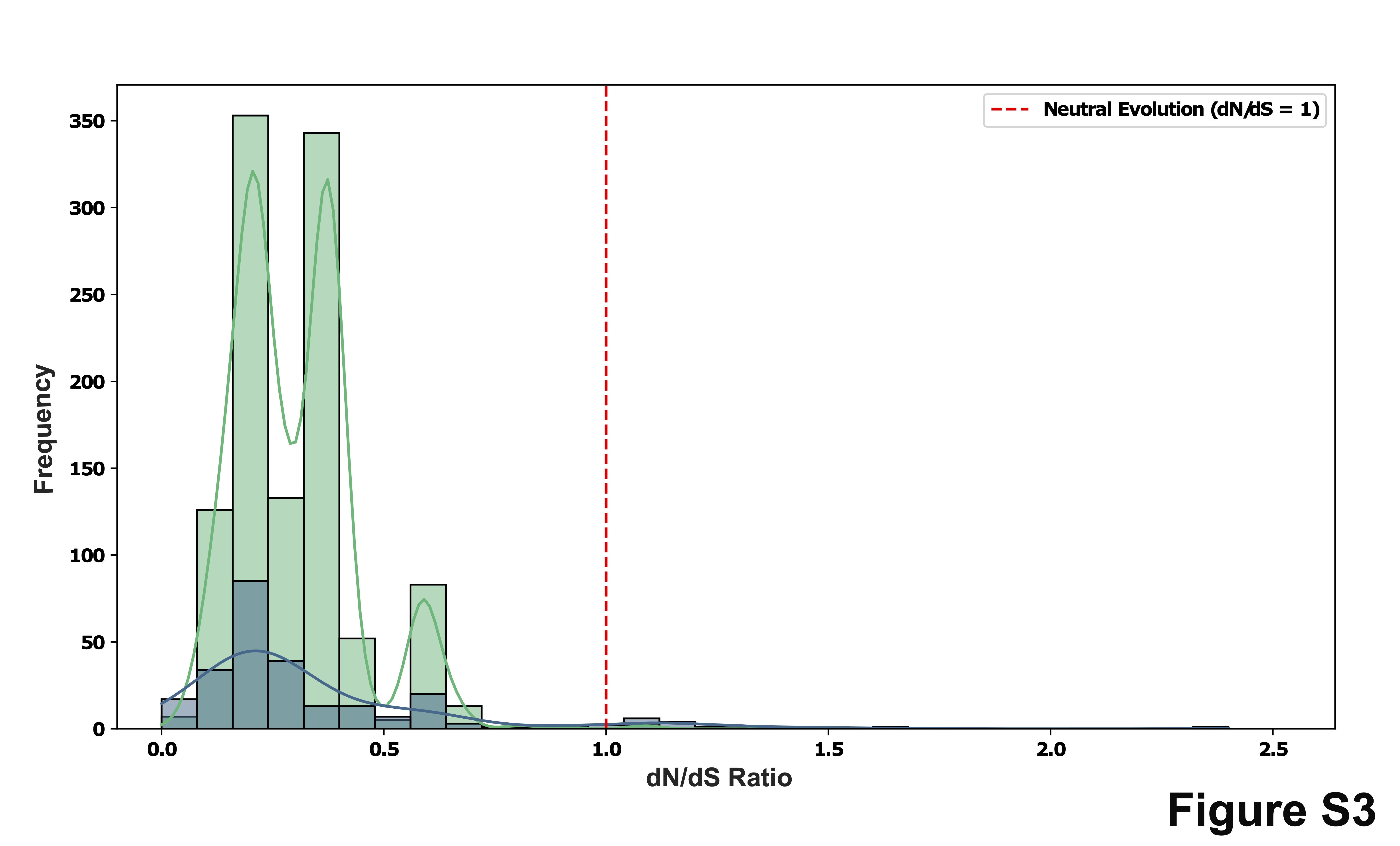

Supplement: Supplementary file 10 — Supplementary Material 10. Figure S3. Distribution of dN/dS ratios. Histograms illustrating the frequency distribution of dN/dS ratios for within-strain and between-strain comparisons. Kernel density estimates are overlaid to show the distribution shape. A dashed red vertical line at dN/dS=1 indicates the threshold for neutral evolution. [file 13100_2025_385_MOESM10_ESM.jpg]
